# Erratum: Lipid membrane-mediated attraction between curvature inducing objects

**DOI:** 10.1038/srep37382

**Published:** 2016-11-21

**Authors:** Casper van der Wel, Afshin Vahid, Anđela Šarić, Timon Idema, Doris Heinrich, Daniela J. Kraft

Scientific Reports 6: Article number: 32825; 10.1038/srep32825 published online: 09 13 2016; updated: 11 21 2016.

This Article contains an error in the Results section under subheading ‘Particle-induced membrane deformation’.

“For this we discern two extreme situations: *tense* vesicles with a (non-fluctuating) spherical shape (*σ* > 1 μN/m) and *floppy* vesicles that exhibit clear fluctuations around a spherical shape (*σ* > 10 nN/m)”.

should read:

“For this we discern two extreme situations: *tense* vesicles with a (non-fluctuating) spherical shape (*σ* > 1 μN/m) and *floppy* vesicles that exhibit clear fluctuations around a spherical shape (*σ* < 10 nN/m)”.

