# Supplementary material for: Lipid membrane-mediated attraction between curvature inducing objects
Source: Sci Rep. 2016 Sep 13;6:32825. doi: 10.1038/srep32825 (PMC5020653; doi:10.1038/srep32825)
Supplement: Supplementary Information [file srep32825-s1.pdf]

Supplementary material for “Lipid membrane-mediated attraction between curvature inducing objects”

Author list: Casper van der Wel, Afshin Vahid, Anđela Šarić, Timon Idema, Doris Heinrich, and Daniela J. Kraft

Supplementary Videos

Videos are separately available online. Here, still images of the videos are shown together with their captions.

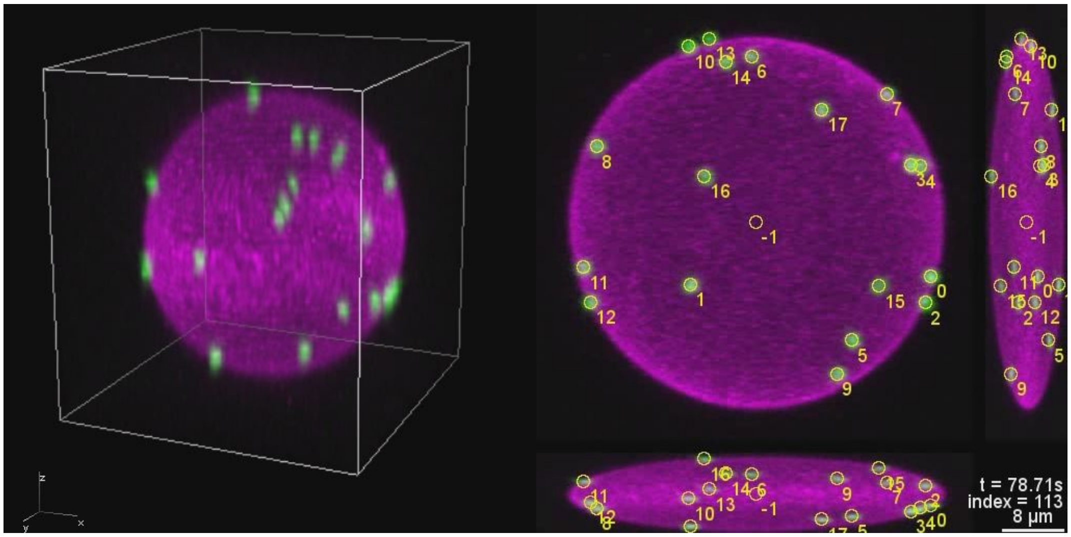

Supplementary Video S1. Three-dimensional confocal image sequence of a Giant Unilamellar Vesicle with attached colloidal particles. GUVs are shown in magenta and particles in green. On the left, a three-dimensional rendering is shown. On the right, the particle tracking is shown in an overlay on three maximum intensity projections (centre: xy, bottom: xz, right: yz). The scale bar denotes the pixel size of the xy projection. Z axes are compressed because the physical size of one voxel is larger in the z-dimension than in the xy dimensions. Label “-1” denotes the vesicle centre. Particles are 1 µm in diameter.

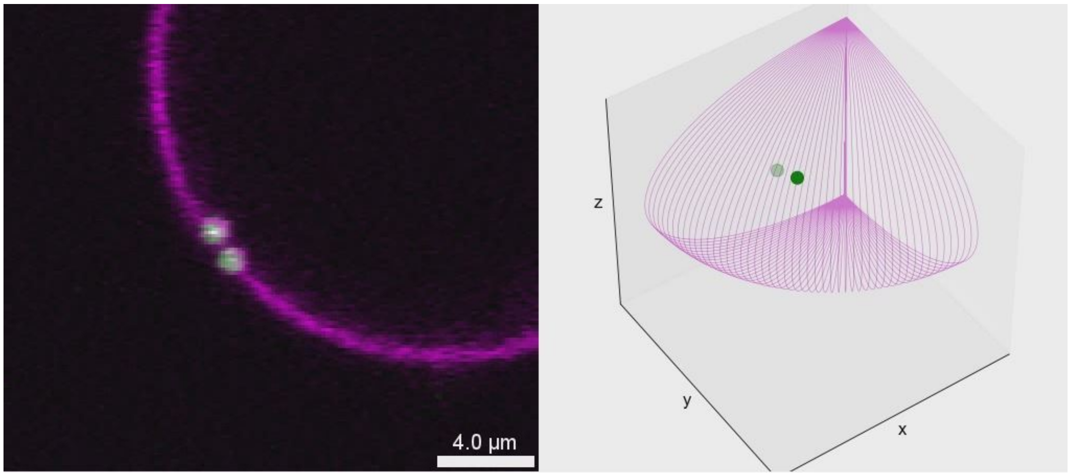

Supplementary Video S2. Three wrapped particles on a tense vesicle. From the particle trajectories, it is clear that the particles attract each other. The video shows an image sequence of confocal slices of a spherical vesicle (in magenta) with colloidal particles (in green), displayed in real time. By using information from the particle-vesicle distance and the vesicle radius, the full three-dimensional coordinates of the particles can be reconstructed. This is shown on the right in a three-dimensional rendering. Particles are 1 μm in diameter.

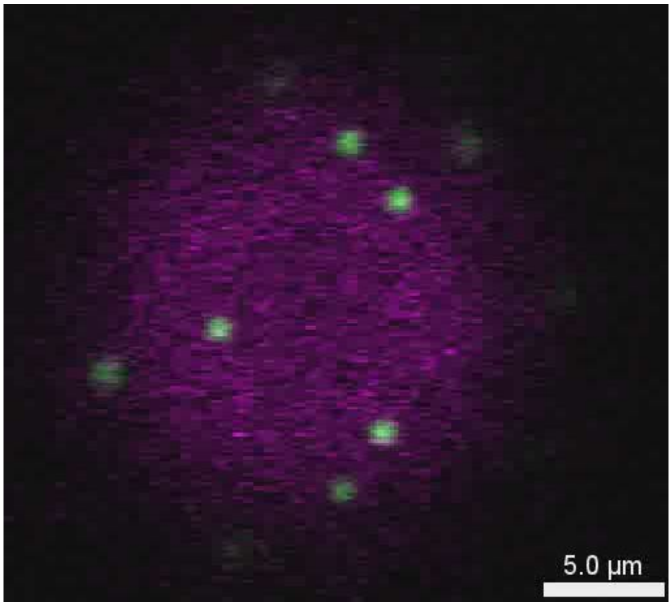

Supplementary Video S3. Non-wrapped particles adhered to a vesicle. The particles do not interact with each other. The video shows a confocal image sequence of the top part of a spherical vesicle (in magenta) with colloidal particles (in green), displayed in real time.

Supplementary Figures

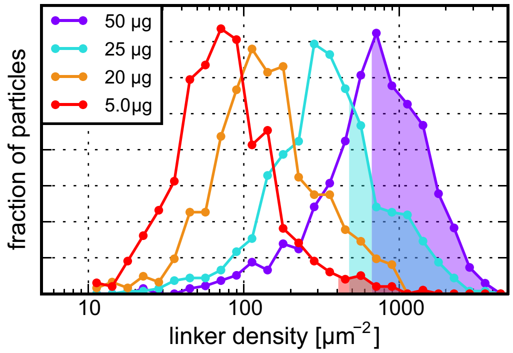

Supplementary Figure S1. Distribution of particle linker densities for four samples with different amounts of linker protein avidin. The linker amount noted in the legend is the amount added to 15 mg particles during synthesis, see Method section. In order to relate the fraction of wrapped particles to these distributions, the right tail of each distribution is shaded up to the measured fraction of wrapped particles. From this, we estimate the critical linker density to be $513 \pm 77\,\mu m^{-2}$. Note that we observed no wrapping for the 20 µg sample (yellow).

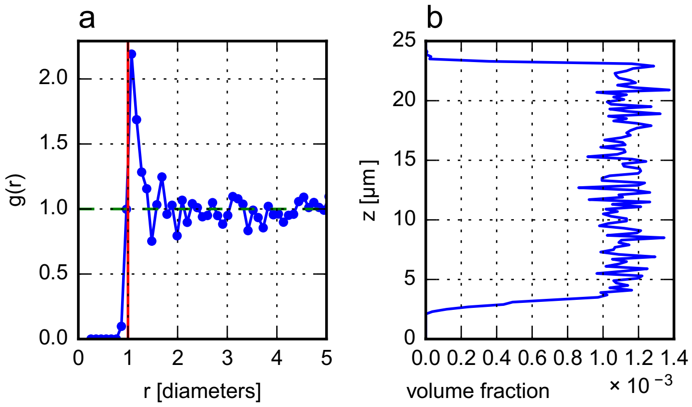

Supplementary Figure S2. Three-dimensional radial distribution function and sedimentation profile of particles suspended at a volume fraction of 0.0011 in a 50 mM density matched PBS solution. (a) The radial distribution g(r) shows no interaction between particles. The red line indicates particle contact. The sharp peak at a distance of 1 diameter is due to the presence of a few dimers originating from the particle synthesis. (b) The density profile shows that there is no gradient in concentration due to gravity.

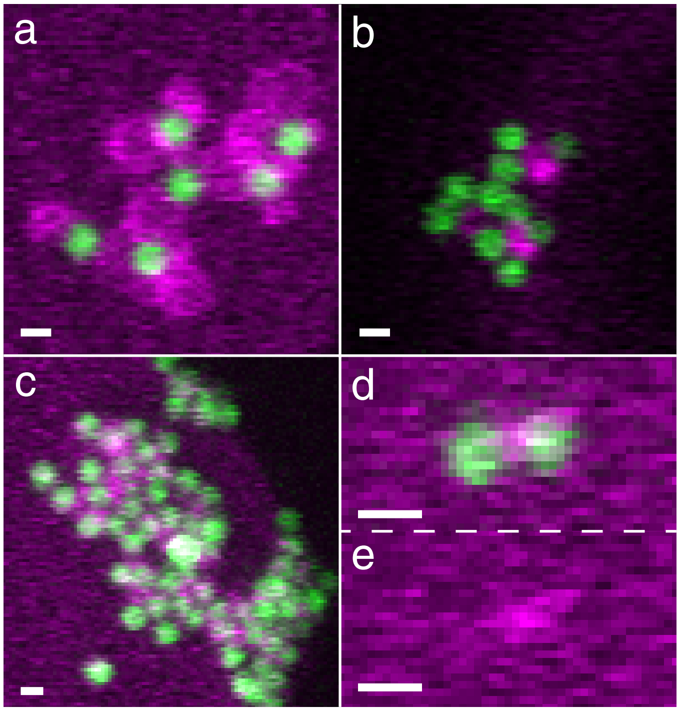

Supplementary Figure S3. Particle aggregates mediated by small lipid structures. In (a)-(c), permanent particle aggregates are shown (in green) that are mediated by lipid structures, that are visible by their fluorescence (in magenta). In (d)-(e) different fluorescence channels from the same permanent dimer is shown, the bright spot of membrane fluorescence in (e) is the lipid structure that causes the irreversible binding. Scalebars are 1 μm.

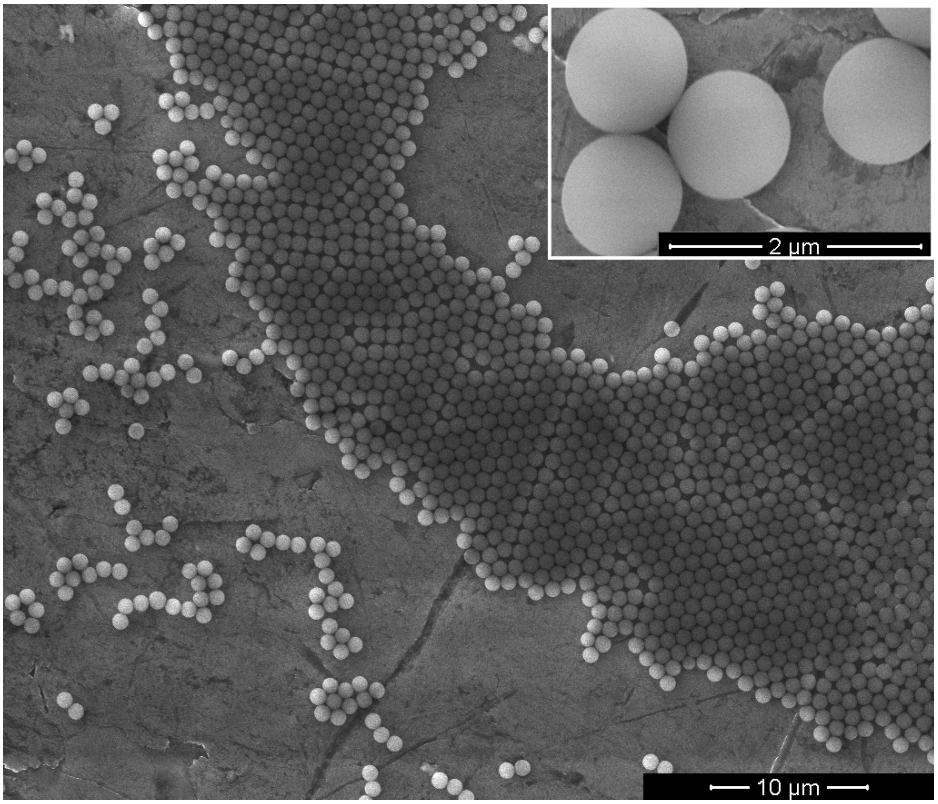

Supplementary Figure S4. Scanning Electron Microscopy image of the 0.98 µm polystyrene colloidal particles used in this work. Images are obtained with an FEI nanoSEM 200 at 15 kV. From the two-dimensional crystallization, it is clear that the size polydispersity is low (0.03 µm). The inset shows the smooth surface of the particles.

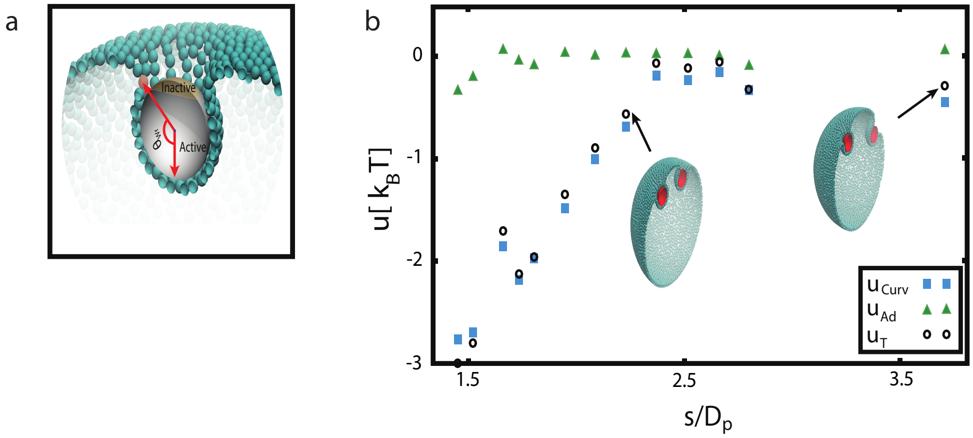

Supplementary Figure S5. Wrapping of particles by the membrane and the resulting total membrane energy in our numerical model. (a) Wrapping happens through adhesion of membrane vertices to colloid particles, due to a strong adhesion potential (Equation 4). We can specify an inactive region at the top of the colloid, preventing the membrane from making very sharp turns (with very high bending energies); in the given example, $\theta_{\mathrm{Wr}} = 11\pi/12$. (b) Curvature, adhesion, and total energy of the system, with zero set at the value of two wrapped particles located at opposite poles of the vesicle. After the wrapping process, the adhesion energy (u_{Ad}) does not change significantly and therefore the curvature energy (u_{Curv}) determines the behaviour of the particles.
